# Cortical Reorganisation during a 30-Week Tinnitus Treatment Program

**DOI:** 10.1371/journal.pone.0148828

**Published:** 2016-02-22

**Authors:** Catherine M. McMahon, Ronny K. Ibrahim, Ankit Mathur

**Affiliations:** 1 Department of Linguistics, Faculty of Human Sciences, Macquarie University, Sydney, New South Wales, Australia; 2 The HEARing Cooperative Research Centre, Carlton, Victoria, Australia; University of Salamanca- Institute for Neuroscience of Castille and Leon and Medical School, SPAIN

## Abstract

Subjective tinnitus is characterised by the conscious perception of a phantom sound. Previous studies have shown that individuals with chronic tinnitus have disrupted sound-evoked cortical tonotopic maps, time-shifted evoked auditory responses, and altered oscillatory cortical activity. The main objectives of this study were to: (i) compare sound-evoked brain responses and cortical tonotopic maps in individuals with bilateral tinnitus and those without tinnitus; and (ii) investigate whether changes in these sound-evoked responses occur with amelioration of the tinnitus percept during a 30-week tinnitus treatment program. Magnetoencephalography (MEG) recordings of 12 bilateral tinnitus participants and 10 control normal-hearing subjects reporting no tinnitus were recorded at baseline, using 500 Hz, 1000 Hz, 2000 Hz, and 4000 Hz tones presented monaurally at 70 dBSPL through insert tube phones. For the tinnitus participants, MEG recordings were obtained at 5-, 10-, 20- and 30- week time points during tinnitus treatment. Results for the 500 Hz and 1000 Hz sources (where hearing thresholds were within normal limits for all participants) showed that the tinnitus participants had a significantly larger and more anteriorly located source strengths when compared to the non-tinnitus participants. During the 30-week tinnitus treatment, the participants’ 500 Hz and 1000 Hz source strengths remained higher than the non-tinnitus participants; however, the source locations shifted towards the direction recorded from the non-tinnitus control group. Further, in the left hemisphere, there was a time-shifted association between the trajectory of change of the individual’s objective (source strength and anterior-posterior source location) and subjective measures (using tinnitus reaction questionnaire, TRQ). The differences in source strength between the two groups suggest that individuals with tinnitus have enhanced central gain which is not significantly influenced by the tinnitus treatment, and may result from the hearing loss *per se*. On the other hand, the shifts in the tonotopic map towards the non-tinnitus participants’ source location suggests that the tinnitus treatment might reduce the disruptions in the map, presumably produced by the tinnitus percept directly or indirectly. Further, the similarity in the trajectory of change across the objective and subjective parameters after time-shifting the perceptual changes by 5 weeks suggests that during or following treatment, perceptual changes in the tinnitus percept may precede neurophysiological changes. Subgroup analyses conducted by magnitude of hearing loss suggest that there were no differences in the 500 Hz and 1000 Hz source strength amplitudes for the mild-moderate compared with the mild-severe hearing loss subgroup, although the mean source strength was consistently higher for the mild-severe subgroup. Further, the mild-severe subgroup had 500 Hz and 1000 Hz source locations located more anteriorly (i.e., more disrupted compared to the control group) compared to the mild-moderate group, although this was trending towards significance only for the 500Hz left hemisphere source. While the small numbers of participants within the subgroup analyses reduce the statistical power, this study suggests that those with greater magnitudes of hearing loss show greater cortical disruptions with tinnitus and that tinnitus treatment appears to reduce the tonotopic map disruptions but not the source strength (or central gain).

## Introduction

Subjective tinnitus is the perception of sound which does not arise from a detectable external physical source. For some individuals, tinnitus may severely affect quality of life, concentration, attention, and working memory [1, 2]. In spite of growing neurophysiological research in humans and animals, the pathophysiological mechanisms that cause tinnitus remain unclear [3–5]. Tinnitus is commonly accompanied by measurable hearing loss [6, 7] or more subtle cochlear pathology without concomitant loss of hearing thresholds, such as outer hair cell dysfunction or disruption of high threshold neural activity [8, 9]. In the undamaged auditory system, spontaneous activity is normally present, but it tends to be relatively weak, incoherent, and masked by background noise [10, 11]. On the other hand, peripheral damage which causes reduced input from the auditory periphery appears to trigger adaptive compensatory shifts in the balance of neural excitation and inhibition that may preserve neural firing rates within a prescribed range. However, an unwanted side effect may be localised hyperactivity or temporal synchrony, resulting in a tinnitus percept (see [12] for a review). Animal models of tinnitus have shown that increases in spontaneous and sound-evoked responses occur in the cochlear nucleus [13, 14], inferior colliculus [15], and auditory cortex [16–18], despite reductions in spontaneous and evoked activity in the primary afferent neurons of the cochlea. Further, in hamsters exposed to loud noise, Kaltenbach and colleagues [14] showed that moderate correlations exist between the peak level of dorsal cochlear nucleus hyperactivity and behavioural correlates of tinnitus, further supporting a more central mechanism of tinnitus.

In addition to central changes in neural activity, tonotopic map reorganisation is widely believed to be associated with tinnitus (see [19, 20]). Map reorganisation, as assessed by neuromagnetic imaging, has been reported in tinnitus patients in whom a measurable hearing loss was present [21, 22]. However, cortical tonotopic map changes have been observed in animals with hearing loss caused by age, loud noise and mechanical disruptions [23, 24]. Therefore, it is unclear whether the association exists between the map disruption and tinnitus percept or the sensory deprivation (or hearing loss).

Multiple studies in humans show the adaptability of the adult brain to auditory training and rehabilitation [25–28], but few have been conducted in the case of tinnitus. Therefore, to better understand the association between tinnitus and disruptions in cortical tonotopic maps and sound-evoked responses, we have measured sound-evoked responses using MEG during a 6 month tinnitus treatment. As hearing thresholds did not significantly change throughout the 6 month rehabilitation process, we assumed that any changes in the source-evoked waveforms and the cortical tonotopic maps, resulted from the tinnitus treatment. We have also compared these responses with a non-tinnitus control group. Specifically, the current study aimed to: (i) identify whether disruptions in the tonotopic cortical maps and source waveform amplitudes occur in adults with tinnitus as compared to those without tinnitus; (ii) evaluate the brain changes of tinnitus sufferers before, during and after a tinnitus sound therapy; and (iii) investigate whether these changes are associated with perceptual measures of loudness and distress.

## Materials and Methods

### Participants

**Fig 1:**
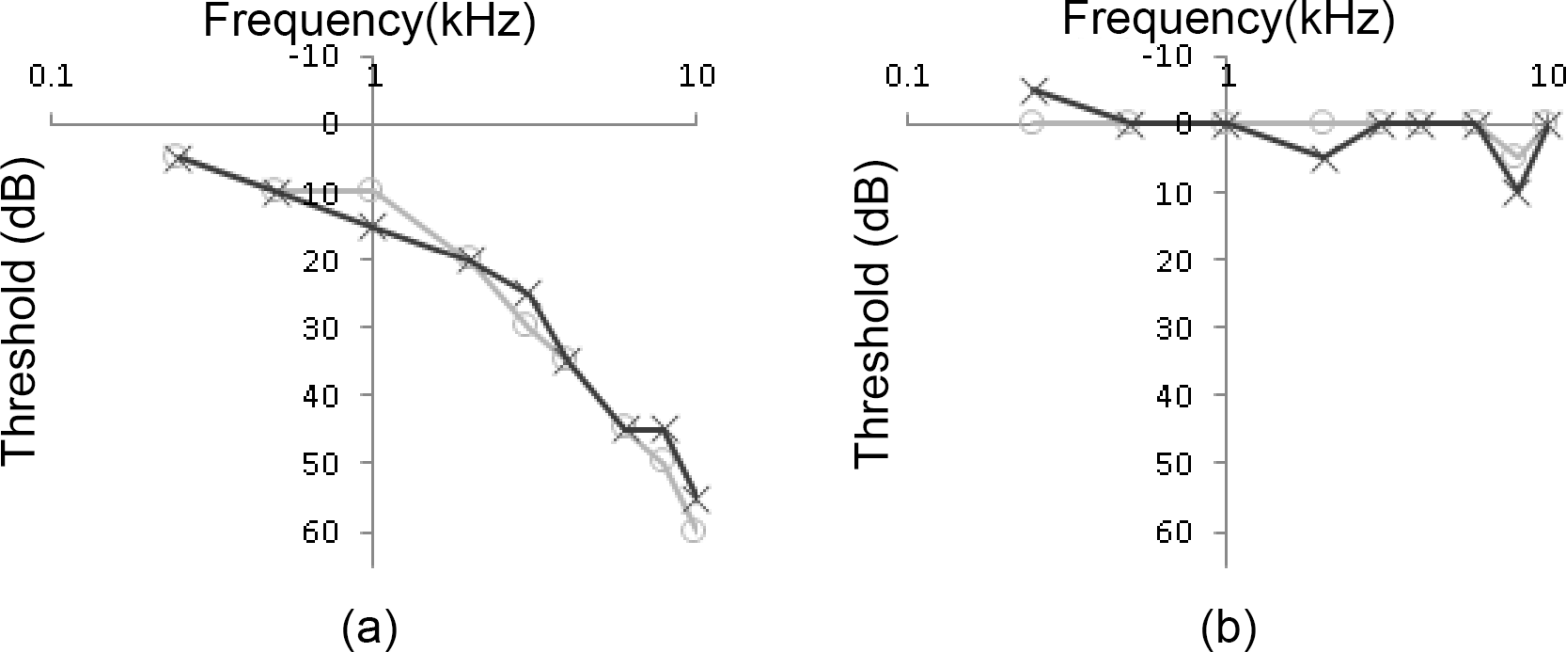
Mean audiograms for the (a) tinnitus group and (b) non-tinnitus group, where the black line represents the left ear and the light grey line represents the right ear.

In this study, 12 bilateral tinnitus sufferers (mean = 54.5 years old, SD = 12.7 years) and 10 normal-hearing non-tinnitus control subjects (mean = 27.6 years old, SD = 5.7) participated. At the time of recruitment, all of the tinnitus group participants reported a history of tinnitus for > 6 months duration, showed high levels of tinnitus distress as measured on the Tinnitus Reaction Questionnaire (TRQ scores ≥17; [29]), had no self-reported clinical depression as measured using the Beck Depression Inventory (BDI scores <29 [30]) or addictive tendencies (WHO-ASSIST < 27 [31]), and had a pure tone average four-frequency hearing threshold PTA_500-4000Hz_ ≤50 dBHL. The mean audiogram of the tinnitus and non-tinnitus control group is presented in Fig 1 and the demographic information on the tinnitus participants is provided in Table 1.

**Table 1:** Subject characteristics.

Participant	Age [years]	Gender	Tinnitus Side	Tinnitus Loudness	Tinnitus Pitch	Tinnitus Duration	Hearing Loss
				[dB]	[kHz]	[years]	
T-01	**59**	M	Equal	12	12	20	M-S^a^
T-02	**56**	M	Equal	9	6	2	M-S^a^
T-03	64	M	Central	5	8	12	M-S^a^
T-04	**58**	M	Left	4	6	1	M-M^b^
T-05	**63**	M	Left	4	6	6	M-S^a^
T-06	**56**	M	Central	8	6	4	N-H^c^
T-07	**24**	M	Right	10	8	8	M-S^a^
T-08	**53**	F	Right	10	4	20	M-S^a^
T-09	**51**	F	Left	8	6	4	M-M^b^
T-10	**42**	M	Central	4	4	3	M-M^b^
T-11	**68**	F	Left	8	6	2	N-H^c^
T-12	**69**	M	Central	6	6	15	M-M^b^

^a^ M-S = Mild–Severe

^b^ M-M = Mild–Moderate

^c^ N-H = No Hearing Loss

Clinical testing, including pure tone audiometry, tympanometry, acoustic reflex testing, and distortion-product otoacoustic emissions (DPOAEs), magnetoencephalography testing, psychoacoustic evaluation of the tinnitus percept (discussed below), and questionnaires (TRQ; Tinnitus Functional Index, TFI [32]; BDI; State-Trait Anxiety Inventory, STAI [33]; Medical Outcomes Study, MOS, sleep scale [34]; SF-36 quality of life measure [35]; and modified self-efficacy scale) were completed at baseline (pre-treatment) and at 5-, 10-, 20- and 30-week points after commencement of the tinnitus treatment program. Psychoacoustic testing included pitch-matching, tinnitus loudness balance, broadband noise (BBN) and narrowband noise (NBN) threshold measurements, BBN and NBN minimum masking levels, loudness discomfort levels, and residual inhibition. Psychoacoustic and self-reported data will be reported elsewhere.

At the baseline evaluation, pure tone audiometry (using air and bone conduction, Madsen OB 822 diagnostic audiometer) was measured for octave frequencies between 250–8000 Hz and at 3000, 6000, 10000 and 12000 Hz using a modified Hughson and Westlake technique. All testing was conducted in a sound proof room. In addition, acoustic reflex testing and tympanometry was performed.

### Tinnitus Treatment

Each of the tinnitus participants completed a 30-week standard Neuromonics Tinnitus Treatment program delivered by an experienced clinical audiologist (see [7, 36, 37] for further information about the remediation program). Briefly, Neuromonics provides a structured program of audiological counselling and clinical support alongside the fitting of an acoustic stimulation device (delivering spectrally enhanced classical music) that is customised to the individuals hearing thresholds. This program was selected because it was highly structured and provided an auditory approach to remediation (rather than a cognitively-based approach). Further, device use was monitored using device logging to evaluate program compliance.

### MEG Testing Procedure

MEG recordings were obtained in a magnetically shielded room using a KIT-Macquarie MEG160 system (KIT, Kanazawa, Japan), which consists of 160 coaxial first-order gradiometers with a 50 mm baseline. Prior to MEG measurements, MEG marker coils were placed on the participant’s head and marker coil positions and head shape were measured with a pen digitiser (Polhemus Fastrack, Cochester, VT). Participants were positioned in a supine position in the MEG environment and pure tone stimuli of 500 Hz, 1000 Hz, 2000 Hz, and 4000 Hz (70 dB SPL; 300ms; 10ms rise/fall) were presented mono-aurally via plastic tubes, with an inter-stimulus interval (ITI) between 900 and 1200 ms. A silent DVD was shown on a projection screen and the participants were told to watch the DVD and ignore the sound stimuli. Block conditions (222 pure tones per condition) were presented for each ear separately in a random order, resulting in a total of 888 pure tones per subject.

### Data Acquisition and Processing

Brain Electrical Source Analysis [BESA] Research 5.3 was used to analyse the neuromagnetic data which were acquired at a sampling rate of 1000 Hz and bandpass filtered from 0.03 to 200 Hz. Adaptive artefact correction which utilised principle component analysis (PCA) was used to remove eyeblinks and heart beat artefacts [38]. Noisy channels (channels which had more than 75% rejected trials) were omitted from further analysis and 1000ms epochs were extracted (including a 400ms pre-stimulus interval). Noisy epochs were rejected (signal channels which amplitudes exceeds 1200fT -this is a stricter epoch rejection criteria compared to that used by [39] to ensure better source modelling) and accepted epochs were averaged to perform source analysis. The aim of performing source analysis was to: (i) determine the neuronal response strengths for each condition; and (ii) detect any disruptions on the tonotopic representation. A montage consisting of eight regional sources was created consisting of six fixed sources and two non-fixed sources, placed symmetrically and bilaterally in the auditory cortex. The aim of using the six additional fixed sources was to ensure that the two sources of interest (that were placed on the auditory cortex region) did not get disrupted from surrounding brain activations, acting as a spatial filter. A time window of 30ms around the peak of the most prominent peak was used for the discrete source search. Source strengths were determined by obtaining the maximum orientation from the source waveform since no substantial activation could be found for orientation 2. The Tailarach coordinate was used to quantify the source location. Subject (T-08) data were omitted from the analysis due to noise. Furthermore, to better understand the relationships between hearing loss and source strength or source location, the tinnitus group was divided into mild-moderate, including the normal hearing subjects (n = 6) and mild-severe (n = 5) hearing loss subgroups based on their pure tone audiogram.

This study was approved by and conducted under the ethical oversight of the Macquarie University Human Research Ethics Committee (ref: 5200900061). Written informed consent was obtained from all participants prior to commencement of the study.

## Results

Ten tinnitus participants showed a high frequency sensorineural hearing loss [ranging from mild-severe], with thresholds between 250–1000 Hz within normal limits (≤20 dBHL), whereas 2 had hearing thresholds within normal limits across all tested frequencies. All of the tinnitus participants had normal tympanograms and middle ear reflexes expected for the degree of measured hearing loss. The tinnitus pitch was predominantly matched to a 6000 Hz pure tone and ranged between 4000–12,000 Hz. Tinnitus loudness was matched between 4–12 dB, using contralateral loudness balance matching.

### N1m Source Strengths and Location comparison between Tinnitus and Non-tinnitus

**Fig 2:**
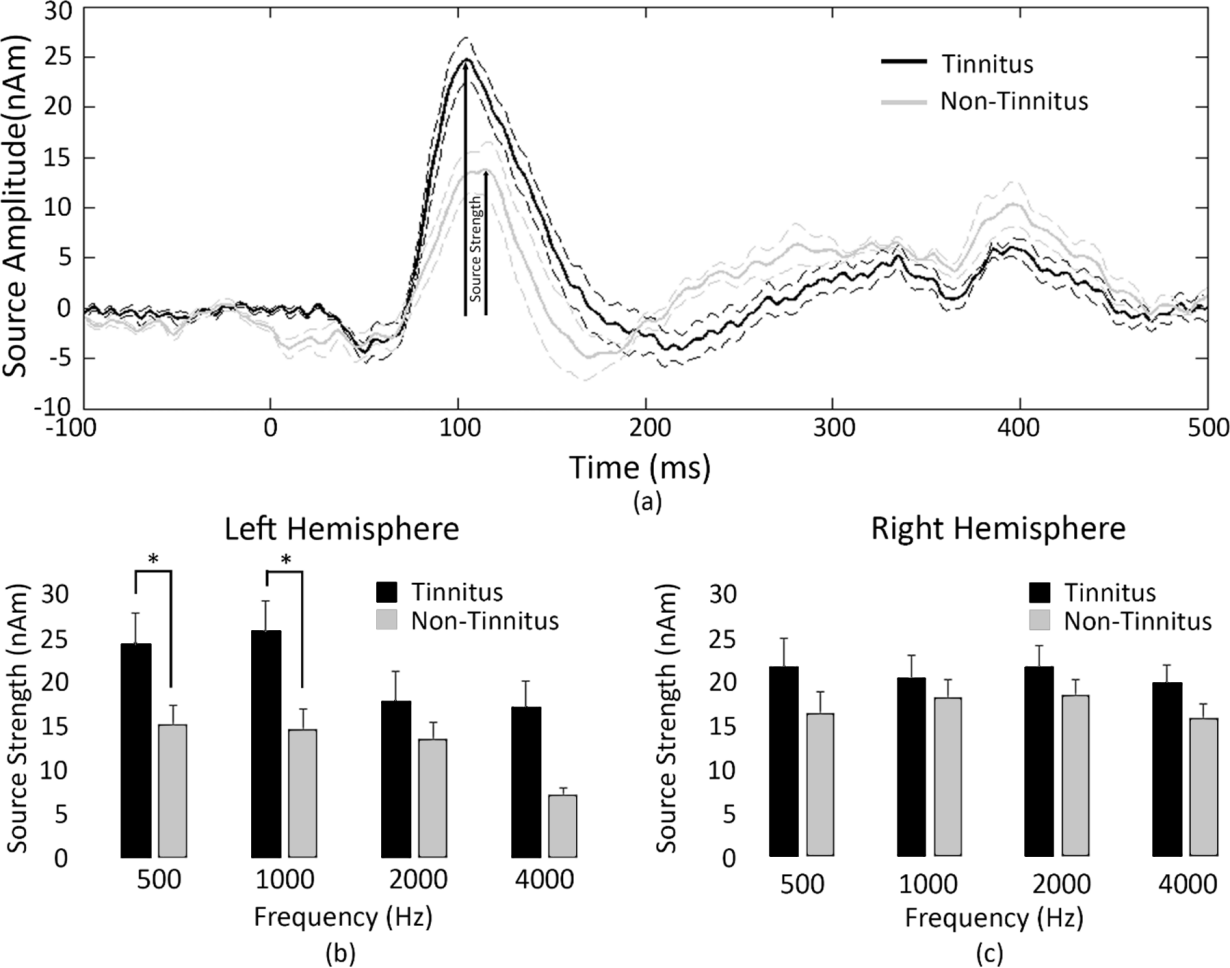
(a) The average 500Hz source waveforms are shown for tinnitus (black, solid curve) and non-tinnitus (grey, solid curve) groups with standard errors shown in dotted lines for the left hemisphere source. (b) Mean (+/-standard error) source strength measured in the left hemisphere in tinnitus (grey) and non-tinnitus (black) groups for octave frequencies between 250–4000Hz. (c) Mean (+/-standard error) source strength measured in the right hemisphere in tinnitus (grey bars) and non-tinnitus (black bars) groups for octave frequencies between 250–4000Hz. Note that asterisks show statistical significance (p<0.005).

A comparison of the left hemisphere mean source waveform is shown in Fig 2A, and mean N1m peak for 500 Hz tone (26.8 +/- 3.6 nAm SE) for tinnitus and (15.2 +/- 2.6 nAm SE) for non-tinnitus participants. N1m peak amplitudes are shown for the left (Fig 2B) and right (Fig 2C) hemispheres for 500, 1000, 2000 and 4000 Hz. Results of a one-way multivariate analysis of variance (MANOVA) with Bonferroni correction showed significantly larger peak amplitudes for tinnitus participants compared with non-tinnitus ([F[4, 14] = 4.137, p = 0.02; Wilks' Λ = 0.458; partial η^2^ = 0.542). There were significant increases in the left hemisphere of the tinnitus participants in the 500 Hz and 1000 Hz frequencies which were tested using a post-hoc univariate ANOVA (p-values of 0.006 and 0.003, respectively) in the left hemisphere but not the right. This difference was not evident at higher frequencies, presumably due to the contribution of high-frequency hearing loss in most tinnitus participants causing a reduction of the peak amplitude of the N1m.

**Fig 3:**
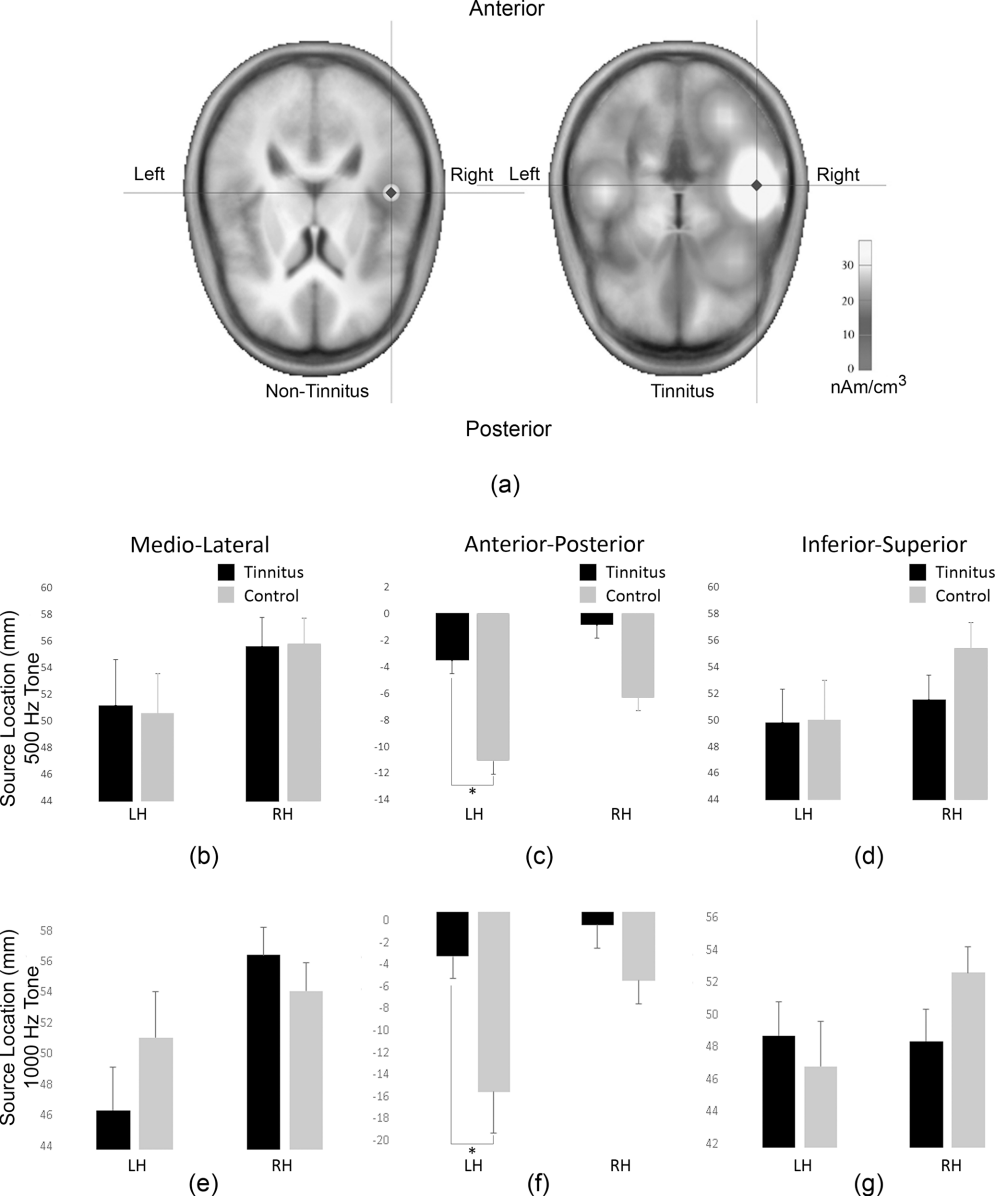
(a) Source location comparison between a non-tinnitus and tinnitus subject on the transverse place from a 500Hz tone (subject T-11). Mean source location comparisons for tinnitus (black bars) compared with non-tinnitus (grey bars) subjects in the (b) Medio-lateral (c) Anterior-Posterior (d) Inferior-Superior plane from the 500 Hz stimuli. Mean source location comparisons for tinnitus (black bars) compared with non-tinnitus (grey bars) subjects in the (e) Medio-lateral (f) Anterior-Posterior (g) Inferior-Superior plane from the 1000 Hz stimuli.

To minimise the confounding effects of hearing loss on the amplitude of the N1m and for comparison between tinnitus and non-tinnitus groups, in following analyses only 500 Hz and 1000 Hz source strength responses were used. Fig 3A shows a comparison of brain dipole modelling for a tinnitus participant compared with a non-tinnitus participant. Disruptions of the cortical tonotopic maps for the 500 Hz source location were investigated by measuring the source location in the medio-lateral (Fig 3B), anterior-posterior (Fig 3C) and inferior-superior (Fig 3D) directions. Similarly, the cortical maps for 1000 Hz were measured and presented in medio-lateral (Fig 3E), anterior-posterior (Fig 3F), inferior-superior (Fig 3G), directions. The mean value of the 500 Hz source location of the tinnitus (mean_ML_ mm 51.0+/-3.3 SE; mean_AP_ -3.7mm +/- 1.8 SE; mean_IS_ 50.9+/-1.76 SE) and non-tinnitus (mean_ML_ mm 49.9+/-2.2 SE; mean_AP_ -11.1mm +/- 1 SE; mean_IS_ 50.0+/-2.2 SE) groups show that the tinnitus group’s source was located more anteriorly than the non-tinnitus group for both left and right hemispheres (Fig 3B), although this was significant for the left hemisphere only (p<0.05). There were no significant differences in source locations in medio-lateral or inferior-superior directions.

### N1m Source Strengths and Source Location during tinnitus treatment

**Fig 4:**
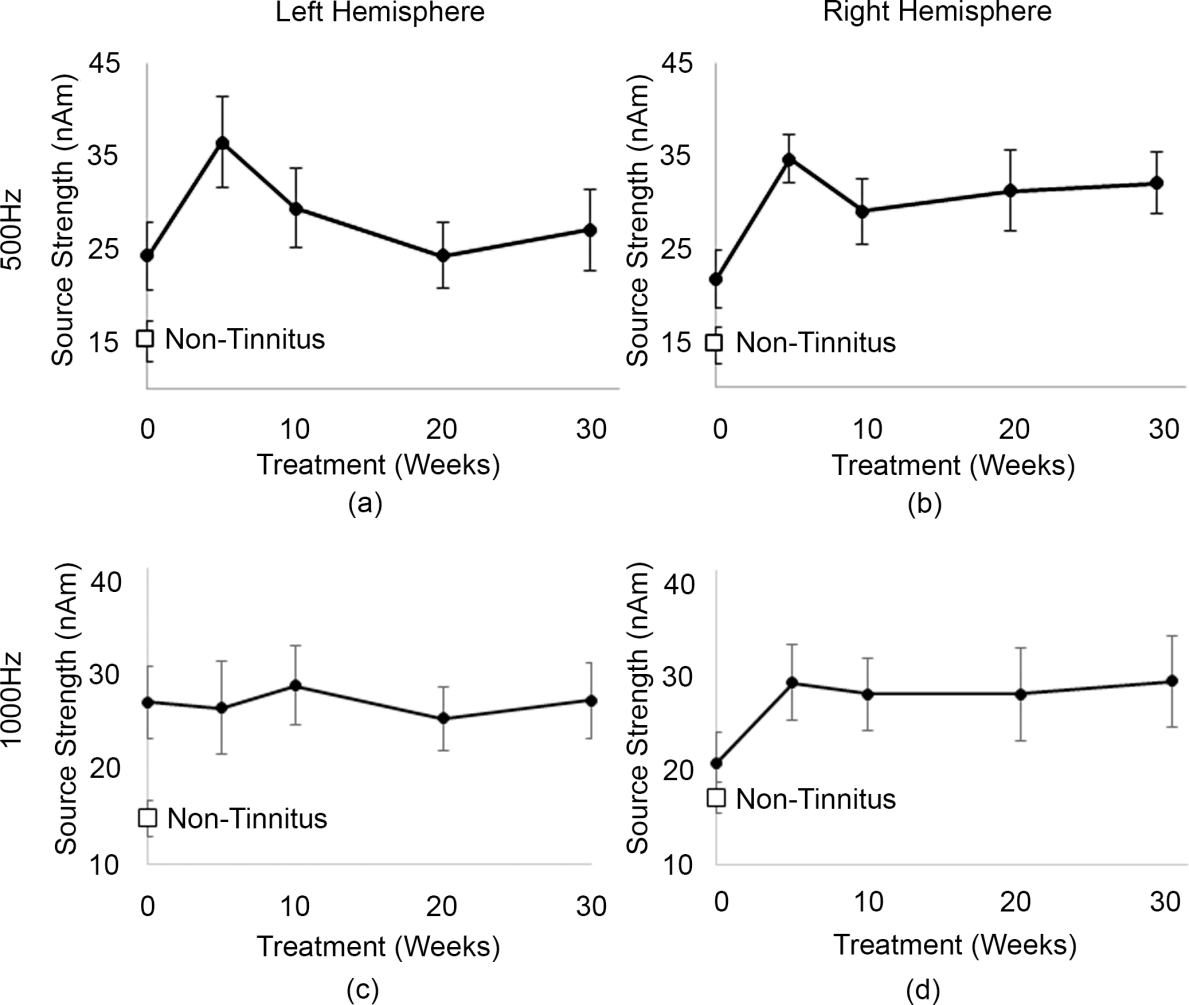
Mean source strength for tinnitus group throughout the 30 weeks tinnitus treatment +/- standard error for (a) 500 Hz tone at the left hemisphere (b) 500 Hz tone at right hemisphere (c) 1000 Hz tone at left hemisphere (d) 1000 Hz tone at right hemisphere. For comparison, the mean +/- standard error source strength for the non-tinnitus group is displayed (white box).

The group average source strengths for the left and right hemispheres for 500 Hz and 1000 Hz throughout the 30 weeks of tinnitus treatment are shown in Fig 4. At baseline, the source strength in the left hemisphere was significantly greater in tinnitus participants than in non-tinnitus participants and remained greater throughout the treatment program. On the other hand, the right source strength amplitude was not significantly higher at baseline, but increased throughout the treatment period, although this was not significantly higher. While the source strength for both hemispheres in the tinnitus participants increased from the baseline testing session to the first test session during treatment (5 weeks after treatment commencement), then decreased at the 10-week test session, a repeated measures ANOVA shows that these changes were not significant throughout the remediation period.

**Fig 5:**
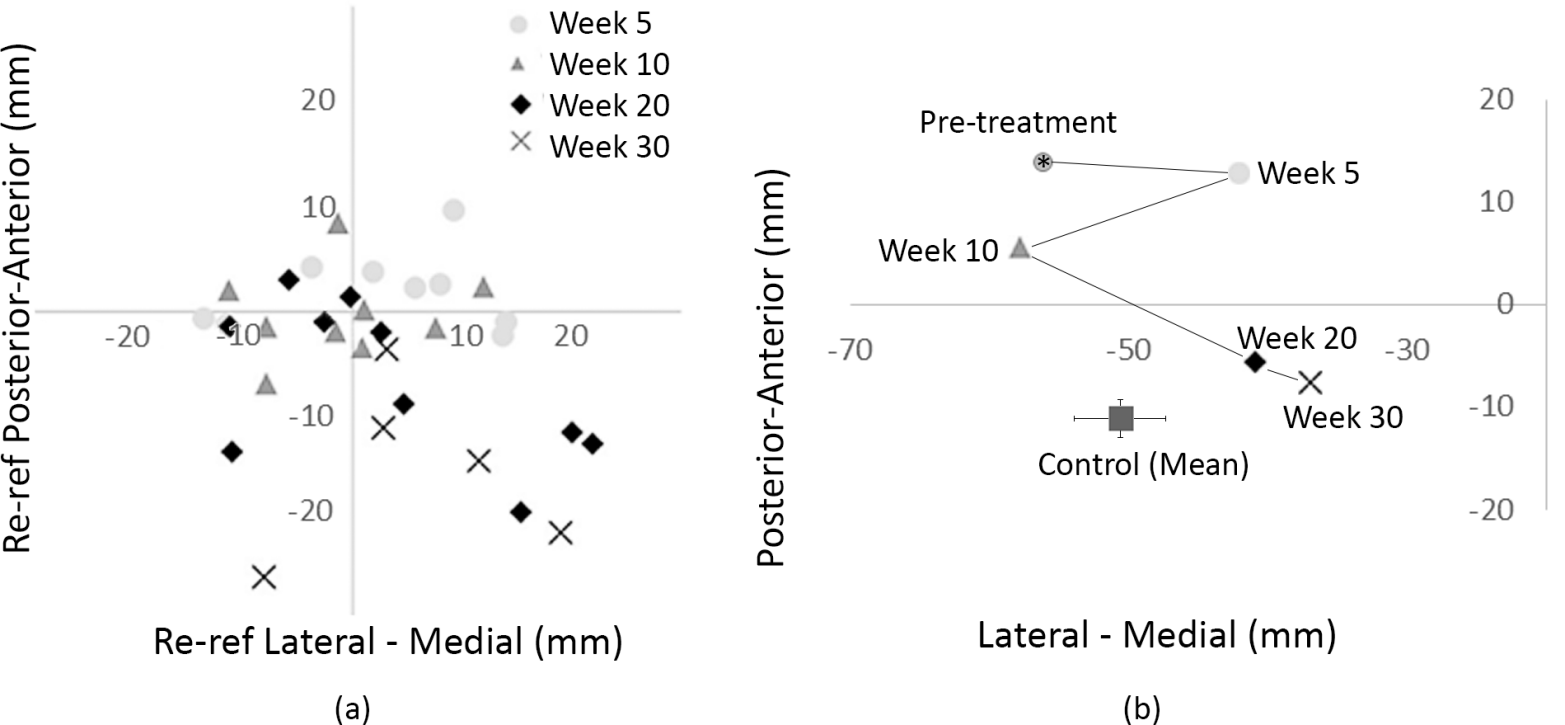
(a) Scatter plot of the left hemisphere 500 Hz source locations for tinnitus subjects referenced to pre-treatment values from week 5–30. (b) Changes in subject T-11’s left hemisphere 500 Hz source location throughout treatment, compared to the mean (+/- standard error) non-tinnitus group 500 Hz source location.

**Fig 6:**
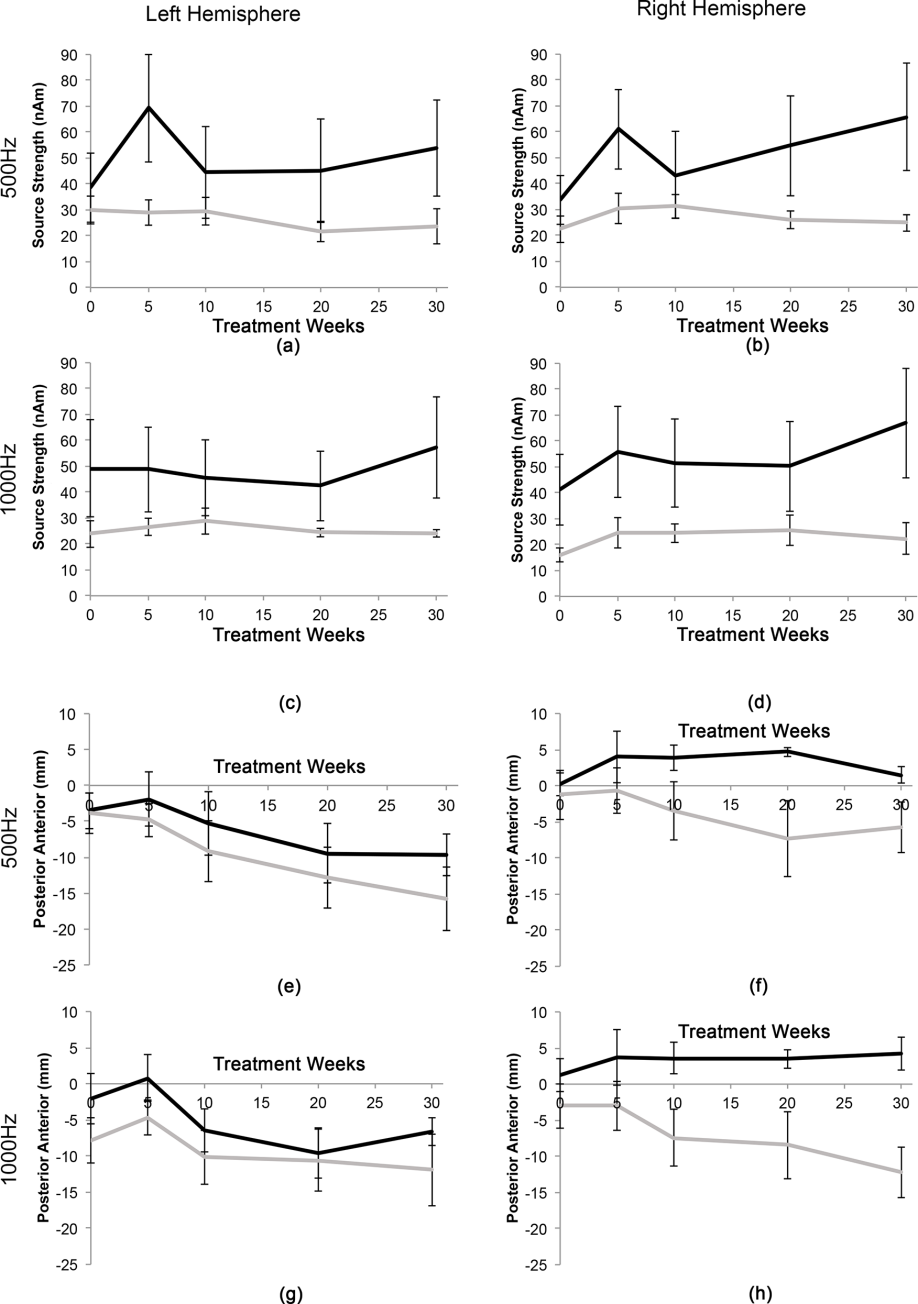
Comparisons between mild-moderate (grey lines) and mild-severe (black line) subjects’ mean (+/- standard error) source strengths for: (a) 500 Hz tone, left hemisphere; (b) 500 Hz tone, right hemisphere; (c) 1000 Hz tone, left hemisphere; and (d) 1000 Hz tone, right hemisphere. Mean anterior-posterior source locations for: (e) 500 Hz tone, left hemisphere; (f) 500 Hz tone, right hemisphere; (g) 1000 Hz tone, left hemisphere; and (h) 1000 Hz tone, right hemisphere.

To determine whether changes in source location occurred during tinnitus remediation, we evaluated the 500 Hz and 1000 Hz sources (in the anterior-posterior and lateral medial direction) pre-treatment and at 5, 10, 20, and 30 weeks during treatment. Fig 5A shows a scatterplot of the source location of tinnitus for all tinnitus participants measured at week 5, 10, 20 and 30 for the left hemisphere. Each data point was re-referenced to each subject’s pre-treatment source location. In general, there was a trend for the source location to initially move slightly more anteriorly (week 5), then more posteriorly (weeks 10–30) over time. The right hemisphere source location displays considerably less movement patterns and is therefore not shown here (see Fig 6 for more detail). In the two participants with normal hearing, similar disruptions and changes to the source strength were observed, supporting Mühnickel and colleagues [21] suggestion that cortical tonotopic map disruptions were related to the tinnitus percept *per se*. However, unlike their study, we only evaluated changes in a non-disrupted frequency regions (500 and 1000 Hz), not the tinnitus frequency and no significant correlations were found between the 500 and 1000 Hz source locations (in any direction—inferior-superior, anterior-posterior, or medial-lateral) and the TRQ score for each participant (data not shown).

To illustrate the movement in source location more clearly, a single participant’s source location (subject T-11) is shown in Fig 5B from pre-treatment to week 30 post-treatment. This shows that the tinnitus source moved towards the control (non-tinnitus) source location (closed square, mean_AP_ -11.06mm +/- 1 SE; mean_ML_ -50.6mm +/- 2.3 SE).

**Fig 7:**
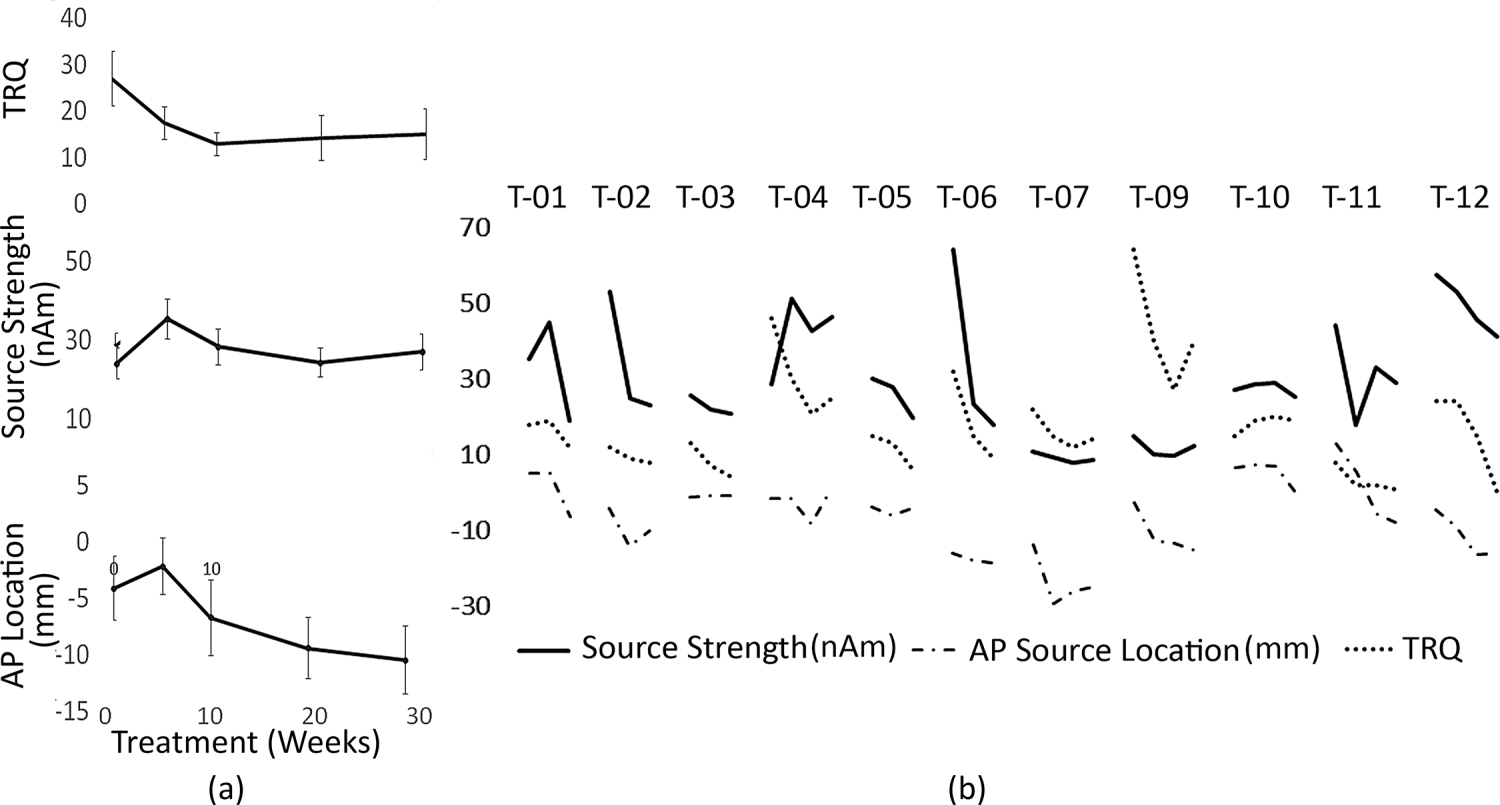
(a) Group mean (+/- standard error) TRQ, source strength and anterior-posterior (AP) location during treatment. (b) Five week time-shifted source strength and anterior-posterior (AP) location and TRQ plotted for all tinnitus subjects.

To determine whether cortical changes measured objectively (source strength and source location) were associated with subjective changes in tinnitus distress (measured using the TRQ), each of these measures were plotted over the treatment time-course (see Fig 7A, where 0 represents pre-treatment). Fig 7A shows that TRQ scores reduce exponentially over the progression of the tinnitus treatment, where the greatest magnitude of change occurs within the first 10 weeks (similar to [37, 40]). On the other hand, the source strength increases, initially, before decreasing and the source location shifts in the anterior direction, initially, before moving posteriorly. Based upon this, to identify whether the trajectory of change for subjective and objective measures were similar for each participant, we have shifted the objective measure results by 5 weeks and aligned and replotted these with the TRQ scores (Fig 6B). It can be seen that the source strength and the source location largely follow the trend for the TRQ score except for participant T-04, which may suggest that psychological effects of tinnitus treatment might precede neurophysiological changes.

To better understand the how hearing loss is affecting the source strength and location, the tinnitus group were sub-categorised into mild-moderate and mild-severe sub-groups. Fig (6A–6D) show the source strengths from the 500 Hz and 1000 Hz tones of the mild-severe group which appear to be greater in left and right hemispheres when compared to the mild-moderate group, however, using a one-way multivariate analysis of variance these were not significant (F[4, 5] = 4.395, p = 0.68; Wilks' Λ = .221; partial η^2^ = .779). Fig (6E–6H) shows the source location comparisons during the 30 week treatment. The posterior source movement (comparing the pre-treatment with the 20-week during-treatment session, where we had the data for all participants) for the mild-moderate group were observed in both hemispheres and frequencies, while the posterior movement of the mild-severe group was only seen on the left hemisphere (Fig 6E and Fig 6G). Using a one-way repeated ANOVA, this was trending towards significance at 500 Hz (p = 0.017) but not at 1000 Hz (p = 0.243). However, the right hemisphere sources (Fig 6F and Fig 6H) do not shift significantly (500 Hz, p = 0.065; 1000 Hz, p = 0.113) over the 20 week time period.

## Discussion

In the current study, we compared mean sound-evoked MEG source amplitudes recorded in response to pure tones at octave-intervals between 500–4000 Hz, and 500 Hz cortical source locations (where hearing loss was not present) in participants with significant tinnitus before, during, and following a 30-week tinnitus treatment program and compared this with a non-tinnitus group. The results demonstrate that significant differences in the mean source strength of 500 Hz and 1000 Hz sound-evoked responses in the left hemisphere exist between individuals with tinnitus compared to those without, which did not change significantly throughout treatment. Further, the mean source location of both frequencies 500 Hz and 1000 Hz were more anterior in the left hemisphere in tinnitus participants at baseline compared to the non-tinnitus group, but no difference was seen along medio-lateral or inferior-superior axes, or for responses in the right hemisphere. Importantly, for the first time in tinnitus participants, we have demonstrated that shifts in the left hemisphere cortical tonotopic map that occur during tinnitus treatment (from 10-weeks post treatment), occurred towards the direction of the source location of non-tinnitus participants. This may provide further support for an association between the tinnitus percept and disruptions to tonotopic maps or could have resulted from sound enrichment (through the Neuromonics device) of areas that have been deprived of sensory input. Finally, we showed that the trajectory of change for self-reported tinnitus distress was similar to the 5-week time shifted objective measures, which may suggest that perceptual changes precede neurophysiological plasticity or that it is the dynamic nature of the change which is more important than the time-course of change.

The difference in the left hemisphere 500 Hz and 1000 Hz source-evoked responses, which were significantly larger in tinnitus participants compared to non-tinnitus participants, is consistent with a model of enhanced central gain [41, 42]. Central gain control has been suggested to play a role in multiple systems, including the enhancement of sensory activity during selective attention [43] and pathological pain [44–46]. Flor and colleagues [47] showed enhanced RMS peak amplitudes of MEG waveforms elicited by electrical bipolar pulses delivered to the back of patients with chronic back pain that were significantly related to chronicity and showed greater activation for painful stimuli compared with non-painful stimuli, presumably mediated by increases in central gain. In tinnitus research, considerable support for central gain has been shown in animal and computational models of tinnitus as well as human studies (see [12, 48, 49]). It could also explain the phenomenon of hyperacusis, which is often associated with tinnitus [50]. Using chronically implanted electrodes in chinchillas, Salvi and colleagues [15] reported that increased sound-evoked responses in the inferior colliculus were measured at high sound intensities after loud-noise exposure, despite reduced responses occurring in the more peripheral cochlea and cochlear nucleus. Brain imaging techniques have also shown cortical hyperactivity in the posterior superior temporal cortex of humans with tinnitus [51, 52], not affected by age or hearing loss. Weisz et al. [20] found significantly enhanced source strength amplitudes at frequencies an octave below the lesion-edge (the audiometrically normal edge of the hearing loss slope) in the right hemisphere in 14 tinnitus participants compared to 11 normal hearing participants, which was correlated with self-reported intrusiveness of tinnitus. Within the current study, there was no significant reduction in the source strength amplitudes during the tinnitus treatment, despite a significant reduction in the self-reported tinnitus severity. There appeared to be a small, but non-significant increase in the source strength amplitudes for both hemispheres at 5 weeks after treatment, which may have resulted from the Neuromonics tinnitus treatment program, which promotes the use of daily sound enrichment within the first 5–10 weeks of rehabilitation. Certainly, if central gain is driven by reductions in neural activity from the periphery, then it is reasonable that modulations to the gain could occur when the neural input is restored or enhanced [i.e. during listening to spectrally enhanced music], but the steady state would be restored once the sound enrichment was removed or significantly reduced. Interestingly, in 8 adults with hyperacusis, Norena and Chery-Croze [50] showed significant reductions in loudness discomfort levels (LDLs) and self-report measures (using the multiple activity scale of hyperacusis) that were retained at least 1 month post-treatment after daily listening to low-level spectrally-shaped pure tones, suggesting a desensitisation of central gain. It is not clear whether the differences in the modulation of central gain between these two studies were the result of differences in treatment paradigm, the pathophysiological problem being treated (i.e. tinnitus versus hyperacusis) or the measures used to assess central gain or simply the result of a relatively small number of participants within each study.

In the current study, significant differences in the amplitude of the source strength were found for low frequencies in the left hemisphere only. The lack of a significant difference in source strength between the tinnitus and non-tinnitus groups for the higher frequencies was likely the result of a difference in the perceptual loudness of the stimuli (which was presented at a fixed level rather than a fixed sensation level), rather than the lack of changes in central gain. Interestingly, the laterality of tinnitus hyperactivity has been demonstrated across multiple studies although the hemisphere which is hyperactive is not consistent across these studies. For example, Positron Emission Tomography [PET] studies using [18^F^] deoxyglucose (FDG-PET) measures in participants suffering from tinnitus show asymmetric metabolic hyperactivity, predominantly in the left hemisphere, independent of tinnitus laterality and handedness, which is significantly correlated with improvements in tinnitus [53, 54].

It is possible that subjective changes in tinnitus severity and loudness perception precede neurophysiological changes that are measured objectively. Certainly in the current study, during tinnitus treatment, the tinnitus participants’ subjective tinnitus distress measure (i.e. TRQ scores) decreased rapidly during the first 10 weeks and then showed no further significant change. On the other hand, objective measurements of the 500 Hz evoked responses showed that the source strength amplitude increased and the source location moved more anteriorly before moving posteriorly towards the location as recorded for the non-tinnitus control group. Similarities between the trajectories of change were observed using a time-shifted comparison between the neurophysiological changes (which occur at a later stage) and perceptual changes (see Fig 4B) where the source moved more posterior after the second treatment session (Week 10). A correlation between source-evoked responses and tinnitus distress might not be a simple relationship, as it may involve other aspects such as attention, stress and emotion [55]. Alternatively, the source evoked responses could be associated with other features of tinnitus, such as the perceptual characteristics, rather than the distress *per se*. Leaver and colleagues [56] have demonstrated that cortical morphological markers of tinnitus distress and perceptual characteristics are not the same. Further, studies in perceptual learning suggest that neurophysiological changes might *precede* perceptual changes, therefore differences in the time course of neurophysiological and behavioural changes are likely to exist across different sensory modalities, although it remains unclear how these interact [57, 58].

Disruptions to the cortical tonotopic map have been observed in humans with hearing loss and tinnitus [21, 59]. Such cortical disruptions typically correspond to the frequencies close to the lesion edge [more clearly observed for steeply sloping hearing losses], where there is an expansion or over-representation of audiometrically normal frequencies adjacent to disrupted frequencies [see [60] for a review]. In the current study, however, the majority of subjects showed only a mild audiometric slope in the higher frequencies, so that a clearly defined lesion edge did not exist, therefore we used a control octave of 500 and 1000 Hz, where hearing was within normal limits [≤ 20dBHL], to evaluate differences between tinnitus and non-tinnitus groups. Consistent with the current study pre-treatment condition, Mühlnickel et al. [21] and Weisz et al. [20] showed that the disrupted frequencies in tinnitus participants were shifted anteriorly, relative to non-tinnitus controls. Specifically, Mühlnickel and colleagues [21] showed in 10 adults with tinnitus and only mild hearing loss that a strong correlation between tinnitus strength and deviation of the tinnitus frequency from the expected tonotopic map existed [r = 0.82, p<0.01] in the contralateral hemisphere. The mechanisms underpinning cortical tonotopic map disruptions in tinnitus subjects are unclear, but may arise from unmasking of intra-cortical or thalamo-cortical connections in the affected frequency region [61]. Interestingly, Norena and Eggermont [62] have shown that disruptions to the tonotopic map in adult cats exposed to loud traumatic noise can be reduced by sound enriched environments. Of fourteen adult cats which were acoustically traumatised, 7 were placed in a continuous sound enriched environment using a high-frequency complex tone for at least 35 days. These cats showed significantly reduced peripheral hearing loss in the higher frequencies and normal cortical tonotopic maps compared to those without sound enrichment, suggesting that the presence of non-traumatic sound can compensate for the reduced neural activity due to hearing loss. In the current study, during the 30 week tinnitus treatment program which used spectrally-shaped classical music, the 500 Hz and 1000 Hz source locations moved posteriorly after week 5, towards the source location of the non-tinnitus participants. Presumably the changes in the source location are a direct result of the sound therapy combined with associated increased arousal and attention [63], rather than reductions in tinnitus impact. Further, subjects with mild-moderate hearing loss showed larger source movements in the direction towards the control non-tinnitus groups which might suggest that they are remediated more effectively over the 30 week time-course evaluated than that of the mild-severe subgroup. However, further studies are needed to determine the relative contributions of the counselling and the sound therapy components of the tinnitus treatment program to the tonotopic map changes.

Brain plasticity in adults during rehabilitation from injury has been observed in other areas of healthcare. Studies in mono-hemispheric stroke rehabilitation using transcranial motor stimulation [TMS] and MEG, show associations between reorganisation of the motor and sensory cortices and functional recovery of limb and hand movements (for a review see [64]). Shifts in the hand motor maps along the mediolateral and anteroposterior axes have been observed [65, 66], as well as hyper-excitability of the unaffected hemisphere, assumed to result from disinhibition [67] and partial restoration of excitability in the affected hemisphere with gains in motor function [68]. For example, Platz and colleagues [69] assessed changes in motor cortex during a 4 week rehabilitation program in stroke patients with severe arm paresis and compared this to functional changes measured using the Fugl-Meyer improvement scores. Using multiple step-wise regression, they showed that a medial shift in the centre of gravity coordinates of the affected hemisphere was the only statistically significant predictor of motor improvement in their model. Understanding brain plasticity might enable new tools for intervention to be designed or better targeted to the individual.

The current study is limited by a relatively small numbers of participants which reduced the statistical power across measures of the longitudinal study, and might limit its generalisability across individuals with chronic tinnitus. Further, given the challenges in recruiting age-matched individuals with similar levels of hearing loss and no tinnitus, the control group was not age- or hearing-matched. However, similar results have been shown in other studies with similar numbers of participants [20, 21], and many of the findings are consistent with animal models of tinnitus, so it is likely that these differences in sound-evoked cortical responses between the two groups are associated with the tinnitus percept rather than the confounders. Further, as hearing thresholds did not significantly change throughout the 30 week treatment program, we assume that the changes in the sound-evoked waveforms resulted from either perceptual changes in the tinnitus or its impact, or a direct result of elements of the treatment program [e.g. sound enrichment].

In summary, the results of our study suggest that tinnitus is associated with increased central gain and disruptions to the cortical tonotopic map and subjects with mild-moderate hearing loss may benefit more from the sound therapy treatment than those with more severe losses. However, while a combined counselling and sound therapy-based tinnitus treatment program might reduce the negative effects of the tinnitus percept and mitigate the disruptions to the tonotopic map, enhanced central gain appears to be maintained, which suggests that it is more related to the reduced sound input from the hearing loss rather than the tinnitus percept per se.
